# Reading the palimpsest of cell interactions: What questions may we ask of the data?

**DOI:** 10.1016/j.isci.2024.109670

**Published:** 2024-04-05

**Authors:** Mihaela Pavlicev, Günter P. Wagner

**Affiliations:** 1 Unit for Theoretical Biology, Department for Evolutionary Biology, University of Vienna, Vienna 1030, Austria; 2 Complexity Science Hub, Vienna 1090, Austria; 3 Yale University, New Haven, CT 06520, USA; 4 Texas A&M University, College Station, TX 77843, USA

**Keywords:** Cell biology, Mathematical biosciences

## Abstract

Biological function depends on the composition and structure of the organism, the latter describing the organization of interactions between parts. While cells in multicellular organisms are capable of a remarkable degree of autonomy, most functions do require cell communication: the coordination of functions (growth, differentiation, and apoptosis), the compartmentalization of cellular processes, and the integration of cells into higher levels of structural organization. A wealth of data on putative cell interactions has become available, yet its biological interpretation depends on our expectations about the structure of interaction networks. Here, we attempt to formulate basic questions to ask when interpreting cell interaction data. We build on the understanding that cells fulfill two general functions: the integrity-maintaining and the organismal service function. We derive the expected patterns of cell interactions considering two intertwined aspects: the functional and the evolutionary. Based on these, we propose guidelines for analysis and interpretation of transcriptional cell-interactome data.

## Graphical abstract

**Figure:**
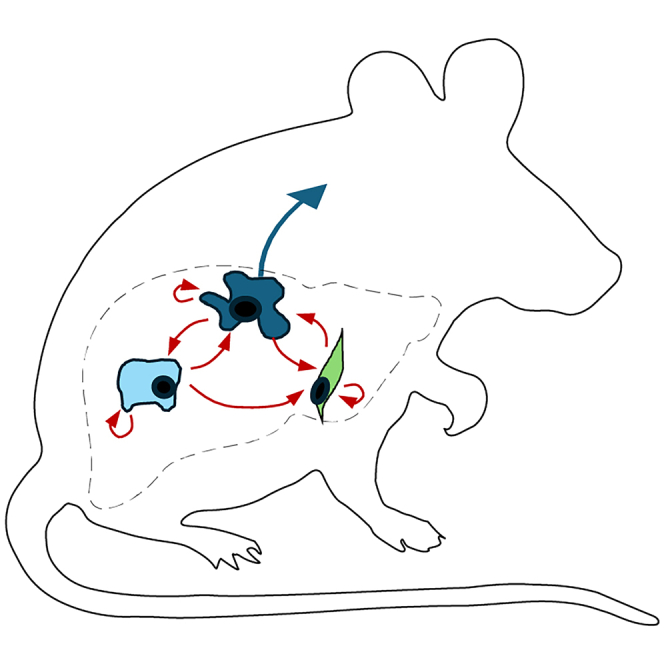

## Introduction

The importance of interactions between parts in constituting the structure and function of organisms has been powerfully illustrated by the physiologist Paul Weiss (1898–1989): an intact chicken embryo has the potential to develop into a chicken. If, as Paul Weiss did for his students, put it into a blender (and switched on), the embryo turns into a mess which consists of exactly the same molecular parts as the embryo, but is not able to develop into a chicken. Structure, arising from past and current interactions between parts (molecules, cells, tissues, and organs), matters to the proper functioning of an organism. We can apply this thinking, in principle, to any level of organization, but to turn the general insight into productive research requires appropriate data. With the development of single cell technology, we can detect potential signaling interactions at scale for many organisms, and a vast array of tools is able to infer the putative cell interactomes from these data.^1^^,^^2^^,^^3^^,^^4^^,^^5^ In this paper, we want to propose a few guiding perspectives to analyze and interpret cell-cell interaction networks.

The focus on cells advocated here has its justification in the degree of autonomy that cells manifest and their ubiquity as an organizational building block. Cells can exist as free living organisms, and even where their degree of autonomy is reduced, as in multicellular organisms, cells manifest their autonomy in the fact that some are surprisingly easily grown in cell culture. Interactions at levels of organization other than the cell have gained much attention: transcriptional regulatory networks and protein interactions have been measured and modeled extensively.^6^^,^^7^^,^^8^^,^^9^^,^^10^^,^^11^^,^^12^ Both levels, even though often without consideration of cellular and other structural context, led to interesting systemic implications, which may become ever more relevant if put into the context of organismal structure.

Beyond Weiss’ early argument cited previously, it is self-evident that many of the cellular functions depend on interactions with or signals from other cells, the extracellular matrix (ECM) and the physico-chemical conditions of their tissue niche, rather than being inherent to the cells (although the potential to interact is dependent on the cell’s competence based on expressing ligands/receptors, secondary messengers or otherwise sensing its environment). Overall, it can be stated that the characteristics of cells are not entirely inherent to the cells themselves but arise also from cell-external factors. Cell external factors consist of those emanating from the cell’s environment, that is other cells, as well as the extra organismal environment. In fact, many extant mechanisms for intercellular communication in multicellular organisms have evolved from mechanisms for environment-sensing in unicellular organisms. For instance, hormone nuclear receptors such as steroid receptors evolved from xeno-sensors responsive to free fatty acids.^13^^,^^14^

Recent decades have seen an upsurge of technological innovations to study processes at single cell resolution. This resulted in numerous cell atlases and even cell interaction atlases available for many tissues, organs and even, in some cases, whole animals.^15^^,^^16^^,^^17^^,^^18^ Interpreting the abundant data on (putative) cell interactions is facing challenges. On the one hand there are the technical issues of how to determine putative cell interactions: especially in non-model species it is difficult to apply the often very promiscuously assembled databases of potential ligand-receptor pairs. Some of the most inclusive databases contain many false interactions, e.g., mistaking co-receptors as ligands. Genomic divergence (e.g., gene paralogs) and the quality of annotation in non-model species further enhance this challenge. Assuming that the technical issues can be overcome, a complementary challenge is the question, how do we extract biologically meaningful information from the “hairball” of putative cell interactions? This is what *palimpsest* refers to; historically, an ever over-written role of parchment, reading from which is used here as a metaphor for extracting crucial information from an abundance of detected and historically contingent interactions (for related use see Hallgrimsson et al.^19^).

We will not address the problem of quality of ligand receptor databases here, beyond stating that critical manual curation is highly advisable when compiling putative cell interactomes; the cell interactions databases may need to be limited to those that are experimentally confirmed (rather than collected by text mining); the interactions with ECM might need to be evaluated separately from secreted ligand-mediated interactions etc. The challenge we want to address here is the second one: the biological interpretation of cell interaction networks. By biological interpretation we mean the analysis of putative cell interactions beyond statistical summaries of the number and pattern of interactions, by integrating the observed patterns into the biological background knowledge.

### 101 on cell interactions: Not all interactions are alike

Before continuing toward our aim, we will briefly summarize the basic cell biology of cell-cell signaling. While all points summarized in the following section are textbook knowledge it is still important to articulate them here, as the summary statistics of “the number of interactions,” more often than not, lumps biologically heterogeneous forms of interactions and thereby potentially confuses more than enlightens the biology at hand.

• Cells communicate with two basic types of signals. At the one hand there are secreted ligands that can reach cells beyond the sender cell’s immediate neighborhood. On the other hand, there are ligands that are embedded in the cell membrane that need physical contact with the receiving cell to reach the cell surface receptors of their immediate neighbors (juxtracrine signaling). In the latter case inference of cell interactions is impossible without anatomical information about the spatial localization of the cell types. In addition, there are secreted but not diffusible molecules that also can interact with cell surface receptors like ECM components (see in the following section).
• One talks of allocrine signaling when the signaling is reaching cells of a different type. Autocrine signaling is to the cells of the same cell type (we will avoid the terms paracrine and endocrine as these relate to the distance over which signals are acting). Cells of the same cell type present the same spectrum of ligands and receptors, and therefore the signaling to the same cell type, not just that to an individual cell, is also considered “signaling to self.”
• The ECM can act as a ligand.^20^ Can we say that the cell that produces ECM components is signaling to ECM attaching cells? Does this form of signaling play the same role as secreted and diffusible signals, like small molecule ligands, say prostaglandins? It is important to clarify this question before engaging in a statistical analysis of cell-cell interaction networks that consistently point to fibroblasts as hub of communication because they produce most of the ECM components. We propose to separate these interactions from those mediated by secreted and diffusible ligands.
• The ECM can act as a repository for secreted ligands. As a consequence, ECM degrading enzymes can cause the release of ligands even if the cell is not producing the ligand itself.
• For all we know, the secreted ligands are anonymous, i.e., they do not have an imprint of their origin. They are perceived by all cells that present the cognate receptor, including the secreting cell (autocrine effects) without recognizing the difference between self (molecules emitted from the same cell type) and non-self (the same ligand emitted from another cell type). This is most certainly the case for small molecule ligands, but peptide ligands are often post-transcriptionally modified and could, in principle, have a cell type of origin imprint (“return address”). We are not aware that this possibility is a biological reality.
• Modulation of signaling can occur by changing the expression (or synthesis) of signal and the receptor, by production or inhibition of decoy receptors or binding proteins, or by the modulation of degradation rate of ligands or receptors.

These basic molecular principles define the complexity of what is often simply summarized as “cell interactions”. We propose that the statistical analysis of any dataset should be stratified by the form and nature of potential interactions to gain biological validity.

### Patterns of cell interactions

No doubt there will be many highly specific expectations for every dataset that are based on the biology of a particular tissue or organ, in a particular species. These are not the kind of patterns that we wish to address here. Instead, we wish to address the theoretical expectations for cell interactions that can be formulated at an abstract level and that may be applicable to cell interaction studies in general. These expectations arise from two intertwined sources: from functional and evolutionary considerations.

#### Functional considerations

Cells either exist as individuals, i.e., as single-celled organisms, or as parts of multicellular organisms. Multicellular organisms come with various degrees of sub-organization of cells, such as tissues, organs, and organ systems. Cnidarians and ctenophores are composed mostly of epithelia, but do not have distinct organs, while on the other extreme vertebrates are composed of many different tissue types organized in spatially segregated organs, like liver and kidneys, which again comprise organ systems, such as circulatory or nervous systems. As these subsystems are themselves specialized for specific functions, such hierarchical organization suggests that cellular functions can be dedicated to two complementary roles (for an excellent analysis see Adler et al.^21^). At the one hand there are activities of the cell types that serve the functional role at the higher organizational levels, such as the tissue, the organ, or the organism. These cellular activities we want to call “service functions” (S-functions). Examples are the secretion of hormones that act at the systemic level, or participation in neural signal transmission. On the other hand, there are activities that are necessary for constituting and maintaining the cells themselves (I-functions for “integrity functions”), and this self-maintenance includes maintaining the organization of the focal level (e.g., tissue) as well as the organization of lower levels, such as the organelles in the case of the subcellular level. We use self-maintenance here to emphasize an active role of interactions; however, the overall result is synonymous with homeostasis.

We can consider these same principles now at the level of tissue: the service functions of a tissue are those that serve the higher levels of organization (i.e., connective tissue serves organ stability), whereas the tissue integrity functions are those that maintain the tissue, and its own organization, that is, they act at the sub-tissue levels. Without working out further examples here, we consider that these principles apply generally across all levels of organization.

Note that the previous “functions” are performed by the interactions of *cells*, even when the function is the self-maintenance or service of a *tissue*, or an *organ*. However, this fact does not justify treating all cell interactions as equivalent for the following reason. The interactions of the cells for the particular tissue level are only possible in the context of the particular tissue, thus are tissue-dependent. More generally, the interactions that are part of I- and S- functions at a specific focal level of organization, require the context of that level. This may provide a way to attribute the interactions to the particular level of organization.

Note also that I and S functions are of course intertwined when we consider an organism as a whole. On the one hand, the I-functions at all levels ultimately also “serve” (i.e., are necessary for) the organism because they enable the conditions necessary to execute S-functions. Along these lines, one could consider the self-maintaining I-function as the “self-service function” for the focal level. Similarly, one could think of the S-functions of all levels as eventually serving the maintenance of the organism as a whole, under whatever external environment it is exposed to. However, this overall resemblance does not invalidate the distinction between S- and I-functions when we study the specific levels of organization. The I-functions are necessary for the S-functions, but the opposite is not necessarily true. This would suggest stronger conservation of interactions involved in I-functions, relative to those involved in S-functions, as will be discussed later.

Two crucial aspects of this proposal thus remove it from a “flat” representation of cell interactions, in which each interaction has an equal weight, making them simply countable. Rather, we propose a structured representation, in which interactions 1- are attributed to distinct levels of organization and 2- differ with respect to their general function in this organization.

To explicate the integration of S and I functions at the level of a tissue, we will make use of a recent theory of tissue organization (Ruslan Medzhitov, personal communication, discussed in DiFrisco et al.^22^). This theory postulates that most tissues consist of a generic mutually supporting core module of four to five cell types from distinct cell categories, regardless of the specific functional role of a tissue. The Medzhitov model suggests that these core cell categories include parenchymatic cells, fibroblasts, macrophages, and endothelial cells. In terms of the model suggested previously, the parenchymatic cells are a cell population that is specialized for an S-function of the tissue, i.e., the functionally specialized cell type of the tissue, such as hepatocytes in the liver. The fibroblasts, macrophages, and endothelial cells instead primarily perform I-functions in the context of the tissue: producing ECM (fibroblasts), monitoring tissue integrity and removing cell debris (macrophages) and ensuring oxygen supply (endothelial cells). Other cells composing the tissue are so-called ancillary cells, specialized for supporting the parenchymatic cells. An example of the latter are Schwann cells for peripheral nerve cells. With respect to the interactions of these cells, the Medzhitov model proposes that the signaling relationships *among* these cell categories, including the parenchymatic cells, are expected to be mostly homeostatic, ensuring the adequate proportion of each of these cells in the tissue and thus an adequate supply of their specific functional contributions. A mutual regulatory relationship between fibroblasts and macrophages, mediated by platelet-derived growth factor alpha (PDGFa) and colony-stimulating factor 1 (CSF1), has experimentally been demonstrated,^23^ supporting the notion that cell interactions *within a tissue module* are, to a large degree, homeostatic. The S-functions of parenchymatic cells are executed by the interaction of these cells with cells *outside the tissue module*, or perform non-communication functions like contraction of muscle cells.

The broad strokes pattern summarized previously is ofcourse a simplification. Fibroblasts and macrophages come in tissue-specific varieties, which also perform service functions in addition to their I-functions. For instance, osteoclasts—specialized macrophages of bone tissue—play an important role in bone remodeling, and lung macrophages clear surplus surfactants from the alveoli, ensuring proper function of the lung. There are also other exceptions: cartilage, for example, consists of a single cell type, the chondrocyte. Nevertheless, the Medzhitov model raises interesting questions with respect to the expected patterns of cell interactions and their role for the organism.

The mutual homeostatic interactions between the cells of a tissue module could conceivably be mediated by a generic set of ligands even in tissues with different S-functions. For instance, PDGFa could be used in any tissue to stimulate the replication of fibroblasts, CSF1 for macrophages, vascular-endothelial growth factor A (VEGFA) for endothelial cells and epithelial groeth factor (EGF) for parenchymatic epithelial cells. As each tissue is spatially contiguous and locally exclusive with respect to other tissues that would suffice to ensure homeostatic maintenance of the tissue core module. To our knowledge, this question has not been addressed on a broad comparative basis across tissues (and species), but could be addressed already, given the abundance of existing data.

Another question that surfaces only when considering that some interactions serve homeostatic functions, is whether the ligands that mediate the homeostatic interactions are always expressed or only when the equilibrium is disturbed. For instance, the expression of VEGFA would only be detectable if the tissue is in a hypoxic state. This means that in a single cell transcriptomic study of an intact (before cell separation) tissue ligand-receptor interaction dedicated to the homeostatic regulation of tissue composition might not be detectable, without contradicting the existence of such a network.

Then there is the question whether the network of homeostatic cell interactions is fully connected, meaning that each cell can stimulate each other cell of the module to either increase or decrease in number if the cellular composition of the tissue is in dis-equilibrium? Or is there a hierarchical structure to the intra-tissue communication maintaining tissue integrity? The former would allow each cell category to call in additional help if it is experiencing some strain, but not necessarily the whole tissue. The latter possibility might allow a more centralized decision-making allowing the integration of various streams of information.

In any case, this way of thinking about cell interactions in tissues challenges researchers to identify the interactions dedicated to S-functions and those necessary for the I-functions in a particular tissue and to identify the network topology dedicated to either.

#### Evolutionary considerations

A dimension that has been less explored so far, is how patterns of cell interactions are shaped in evolution and thus what is to be expected when we compare interaction networks between species. This is our aim in this section. We develop several hypotheses about the evolution of patterns of intercellular interaction.

##### Internal, in addition to external selection

As aforementioned, the service and integration functions of cell interactions are under different evolutionary constraints. S-functions are more guided and directed toward the environmental adaptive needs: those arising from the external environment, in part via higher organizational levels. I-functions are shaped to ensure the integrity of the tissues and organs. S- and I-functions are thus roughly subject to external (adaptive) selection and internal selection, respectively.^24^^,^^25^^,^^26^^,^^27^^,^^28^ Given these broad patterns, the cellular interactions underlying S-functions are likely more variable among species and certainly among parts of the organism, i.e., are specific to tissue and organ types, because they define the functional role of the respective higher-level unit (tissue or organ, or body part). In contrast, the I-functions and the underlying cellular interaction patterns are likely more conserved and generic as they ensure the existence and integrity of the higher level unit, independent of the specific S-function performed. However, if the I-functions are redundant, systems drift could cause differences between species, i.e., changes to the network that do not affect functionality.

##### The double contingency constraint

A signaling interaction between two cell types, say X and Y, is the result of two evolutionarily contingent outcomes: the expression of a ligand in cell type X and the simultaneous expression of the cognate receptor in cell type Y or vice versa. This obvious fact has important implications, namely that the expression of ligand and receptor is contingent, i.e., there is no mechanistic necessity that either is expressed in a particular cell type. Because of this, the *de novo* evolution of a signaling relationship is unlikely, as the two scenarios briefly illustrate in the following text.

The first scenario is that two independent mutations, one causing the expression of the ligand specifically in cell type X and the other causing the expression of the receptor specifically in cell type Y, occur simultaneously to produce a functional signaling relationship. Each mutation separately would be functionally irrelevant or even costly, as the cell would waste energy to produce a useless product. The only way natural selection could be able to pick up on such a *de novo* interaction is if these two mutations occur simultaneously in the same individual. If they occur in different individuals in the same population, the likelihood that they combine, by random mating, in the same genome of a diploid species is initially (1/2*N*_*e*_)^2^, with *N*_*e*_ being the effective population size. The likelihood of recombining into the same genome (and thus becoming functionally relevant) is thus initially very low. An alternative scenario would be for a single mutation to change the expression of the ligand in X and the receptor in Y simultaneously. We are not aware of a molecular mechanism that would make such a mutation plausible. Hence, the evolution of functionally relevant cell-cell interactions is more likely to occur through evolutionary steps with single mutations causing the expression or repression of either a ligand or a receptor in a particular cell type or cell type family. This principle severely constrains the plausible evolutionary scenarios for the evolution of a cell-cell communication network. One such scenario is the pruning of signals discussed in the next section. Pruning only requires the removal of one of the two components (ligand or receptor) to interrupt and thus re-shape the interaction network. Another scenario is “listening in”: introducing a receptor on a cell that allows receiving signals from a cell that already expresses the cognate receptor (ligand). This scenario would first extend the network, but may also be followed by the subsequent pruning, leading to rewiring of the network.

#### Predicted consequences of the functional and evolutionary considerations for the expected structure of cell interaction patterns

##### Evolving cell type identity by pruning of autocrine interactions

Cells are the building blocks of multicellular organisms, and correspondingly, organismal complexity is often estimated as the number of distinct cell types in an organism.^29^^,^^30^^,^^31^ A relevant evolutionary question in this context is how do novel cell types originate? A broad-based effort to address this question is underway by documenting the phylogenetic origin of cell types and the underlying mechanism in terms of the intrinsic gene regulatory logic of cell type identities.^32^ However, given that cell types play specific roles in the broader organismal context, an important aspect of their origination is how new cell types are integrated into the network of cell-cell interactions in a tissue or organ.

To address this question, one can think of the possible cell interaction networks as lying on a spectrum between two (theoretical) extremes: on the one side is an aggregate of cells of the same type, and on the other an organism with as many cell types as there are cells, possibly approximated by “eutelic” animals, i.e., small animals with constant cell numbers, like nematodes and rotifers, in which each cell performs a different essential function.^33^ In an aggregate of identical cell types, all cells of the same cell type are producing the same ligands and receptors, and therefore all intercellular signaling is autocrine and ubiquitous. In the other extreme, the cell types never express receptors for ligands they themselves produce. In this case all interactions would be allocrine.

**Figure 1:**
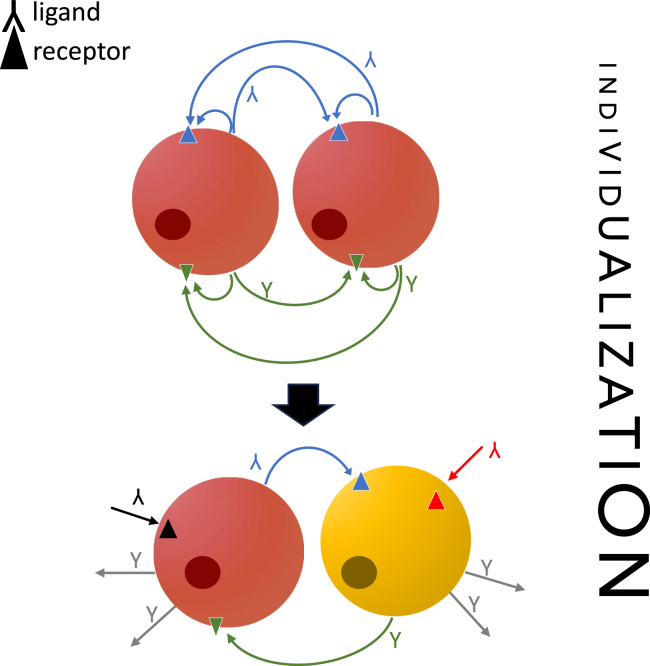
Cell individuation As the cells diverge in evolution, signaling to other cells is not anymore identical to autocrine signals. The autocrine signaling thus diminishes and is replaced with allocrine signaling.

From the previous perspective, the divergence between cell type identities would be reflected in the divergence between the spectrum of ligands emitted and signals that can be received. In this process, autocrine interactions are transformed into allocrine ones by pruning autocrine mediators, i.e., differentially shutting down the expression of ligands and/or receptors or expressing new signal mediators in a subset of the cells (Figure 1). By pruning, a set of cells that originally were of the same type, expressing the same ligands and receptors, are turned into two different cell types with directional allocrine signaling between sister cell types. To test this, one could expect that the pattern of ligand-receptor pairs in sister cell types reflects the full autocrine signals from the ancestral cell types. This would be detectable by comparing the ligand-receptor spectrum of sister cell types with that of the homologous single cell type in an outgroup species.

One proxy to measure where a cell type is on this spectrum of individualization, is to determine the proportion of allocrine signaling compared to the total signaling a cell type engages in. That is, the proportion of receptors, out of all expressed receptors, for which the signals are not produced by the cell type itself, and the same for the ligands. These proportions can be expected to increase in the process of cell type divergence and autonomization. One may thus distinguish individualized cell types that to a great extent rely on allocrine signaling, from less individualized cells in which a great portion of cell signaling is potentially autocrine (but see next section, discussion of migratory cells).

##### Adapting to the instability of tissue context by pruning of allocrine inputs

The cell type-specific profiles of ligand and receptor expression may be expected to evolve dependent on the temporal stability of a cell’s tissue context. Specifically, migratory cells, such as leukocytes, are exposed to a wide range of ligand environments over time, and therefore to a wide range of ligands. One would expect these cells to reduce the expression of receptors, limiting them to those involved in the cell type specific S-function and thus respond to only “service-relevant” signals. Migratory cells are thus also not expected to engage in tissue maintenance as are tissue resident stromal cells. Exposure of the cell to variable environments may thus drive a reduction of the cell’s receptor inventory to S-function-relevant signals. In contrast, the tissue-resident cells encounter a less variable signal environment and may therefore be able to express a less fine-tuned spectrum of receptors, overall, including some for which the ligand is simply not present, or that would be too promiscuous in a more stringent context. An interesting system to test this idea would be a comparison of the receptor inventory of peripheral and tissue resident members of the macrophage cell type family. In addition, the role of these interactions of tissue resident cells may be more focused on tissue homeostasis and being more redundant rather than performing specific service functions for the organism. Redundancy among these interactions would also predict that these interactions are readily subject to developmental systems drift,^34^^,^^35^ i.e., differences between species that do not affect the function and identity of the tissue.

##### Evolution of novel spatial relationships of cells and tissues is constrained

During embryonic development and during evolution, the spatial relationship and thus the potential for signaling between cells in tissues, can change. In particular in early development, induction via signaling between tissues is common. For instance, during the development of the amphibian embryo, the epidermis that eventually develops the lens of the eye interacts with a variety of cell populations, including pharyngeal and heart mesenchyme, in addition to the eyestalk.^36^^,^^37^

In evolution, epidermis can contact different kinds of dermal tissue with implications for the epidermis differentiation. For instance, whether rodents form cheek pouches lined with mucosal tissue or fur depends on the location of the cheek pouch invagination point and thus to what dermal tissue the pouch epithelium is exposed.^38^ Another dramatic novel tissue contact is the evolution of placentation, where extra-embryonic tissues of the embryo get into contact with the uterine endometrium, if/when the eggshell is lost.^39^ A question to ask is, how likely are new spatial relationships compatible with the cell-cell signaling relationships in each of the participating tissues?

The aforementioned model by Medzhitov suggests that the cell type categories (e.g., parenchyme or macrophage) occur repeatedly across tissues. If so, can we expect that the new cell interactions will more readily be accommodated if the involved cell categories have evolved in a similar tissue context, such as epithelial cells that have been in contact with stromal cells and become introduced to a different stromal cell type at a new interface? We would thus expect that such evolutionary changes would be more often successful and represented in phylogenies leading to the extant species. This could present another way how the present organization influences the likelihood and kind of evolutionary change. A potential example in which to ask this question is the aforementioned origin of the maternal-fetal interface in mammalian viviparity. In mammals with hemo- or endotheliochorial placentation the uterine luminal epithelia are eroded during the placental development and replaced with placental (=epithelial) cells, resulting in the new maternal-fetal tissue unit.

## Conclusion

In this manuscript, we attempt to provide an organismal perspective of cell communication, informed by functional and evolutionary considerations, which may guide our further analysis of cell interactions.

• Trying to distinguish between subnetworks for service (S-) and integrity (I-) functions might help focus attention to relevant parts of the network. For instance, degenerative diseases are likely due to failure of I-functions, while organ function diseases (renal failure) could be due to both, failures of I functions as well as S-functions, because I functions are necessary for performing S-functions but not the other way around.
• Scenarios for the evolution of cell interaction networks should respect the double contingency condition, i.e., the unlikely *de-novo* origination of the expression of a complete ligand-receptor pair in appropriate cell types. Scenarios based on single step deletion or expression of ligands or receptors are more likely, as for instance the pruning of an autocrine network of interactions into allocrine interactions, or duplication and divergence of ligand or receptor, or evolution of receptors for existing molecules not previously acting as ligands (e.g., ligand exploitation model^40^).
• Evolutionary changes in the spatial relationship between tissues can lead to novel interaction patterns, as is the case in the evolution of the placenta, where maternal and fetal tissues come into contact.
• The receptor inventory of a cell might reflect the stability of the cell’s environment with more migratory cells being more restrictive in receptor expression than tissue resident cells.

The elementary considerations also suggest two technical recommendations for the analysis of putative cell interaction networks inferred from single cell transcriptomes.

• First, the analysis of putative interactions should be stratified by their molecular biology (paracrine, juxtacrine, ECM-mediated, etc.). Different molecular types of interactions reflect different kinds of biology and shall not be lumped into summary statistics.
• Second, the complementarity of ligands and receptors in a dataset may serve as a hint about data quality. If there is a strongly expressed ligand that has no local receptor, it is either a hormone, or the expression of the cognate receptor might have been missed.

The value of any new concept lies in the productive research questions that it opens. The present conceptualization of cell-interaction structure calls for ways to distinguish the interactions that fulfill the two types of functions, the service, and the integrity functions, at a given level of organismal organization. These tasks are not simple to accomplish and require an organismal biological view. We attempt here—next to the evolutionary hypotheses—to provide some ideas and specific questions throughout the paper, suggesting how these challenges may be tackled.
